# Marked seasonality and high spatial variation in estuarine ciliates are driven by exchanges between the ‘abundant’ and ‘intermediate’ biospheres

**DOI:** 10.1038/s41598-017-10308-y

**Published:** 2017-08-25

**Authors:** Ping Sun, Liying Huang, Dapeng Xu, Bangqin Huang, Nengwang Chen, Alan Warren

**Affiliations:** 1 Key Laboratory of the Ministry of Education for Coastal and Wetland Ecosystem, College of the Environment and Ecology, Xiamen University, Xiamen, 361102 China; 2 State Key Laboratory of Marine Environmental Science, Institute of Marine Microbes and Ecospheres, College of Ocean and Earth Sciences, University, Xiamen, 361102 China; 3 Department of Life Sciences, Natural History Museum, London, SW7 5BD UK

## Abstract

We examined the spatial and temporal variability of ciliate community in a subtropical estuary by rRNA and rDNA-based high throughput sequencing of 97 samples collected along the entire salinity gradient at two-month intervals in 2014. Community divided statistically into three groups: freshwater (salinity < 0.5‰), oligohaline and mesohaline (0.5‰ < salinity < 18‰), and polyhaline and euhaline (18‰ < salinity < 40‰). Across all three groups, salinity explained most of the community variability. Within each group, seasonal shifts in community formed cool (spring and winter) and warm (summer and autumn) subgroups, indicating that spatial variability overrode seasonal changes in determining community composition. Cool and warm groups showed opposite associations with temperature and prey proxies, suggesting distinct seasonal niche separation. The community reassembly of cool and warm groups was essentially due to transitions between intermediate (with relative abundance of 0.01–1%) and abundant (with relative abundance > 1%) OTUs. Further analyses demonstrated that the intermediate group not only encompassed comparable OTU richness to that of the total community and maintained high metabolic activity but also had the highest proportion in transition, either to abundance or rarity, thus offering a first view on how it varies across space and time and revealing the essential role it played in maintaining stability and functionality within the community.

## Introduction

Microzooplankton are heterotrophic organisms with body size between 20 and 200 µm. They can feed on pico- and nanoplankton unavailable to most meso- and macrozooplankton. They therefore occupy a key position in aquatic foodwebs as their grazing significantly affects primary producers and bacteria^1, 2^. However, our knowledge of the spatial and temporal distribution patterns of microzooplankton across a wide variety of environments and their shaping factors is still limited. Ciliates are single-celled eukaryotes (protists) that dominate many microzooplankton communities in terms of both species diversity and abundance^3^. By virtue of their short generation times, ciliates can quickly respond to environmental fluctuations^4^. Therefore, understanding the community dynamics of ciliates is increasingly important in an era of rapid environmental change, particularly in habitats where such changes are likely to be sufficiently pronounced to affect ecosystem function. Estuaries are highly dynamic ecosystems that undergo constant change due to both natural, e.g. daily (tidal) and seasonal variations, and anthropogenic factors. These variations in space and time make estuaries ideal ecosystems to perform investigations targeting the community dynamics of ciliates.

Estuarine ciliates have been included as part of investigations on wider taxonomic scales, e.g. protozoa^5^ or protists^6^, or alongside studies of various metazoas groups, e.g. copepods, fishes, molluscs and crustaceans^7^. Other studies have focused on specific groups of ciliates e.g. tintinnids^8^ or aloricate ciliates^9^. Studies on the full spectrum of ciliates along entire salinity gradients are limited, and those dealing with both temporal and spatial variations are even rarer^10, 11^. Furthermore, most of these studies were done with classical methods, e.g. fixing water samples with Lugol’s or Bouin’s and directly identifying and/or counting species under the microscope. There is increasing evidence, however, that convergent or plastic morphologies can mask major molecular, physiological and ecological differences, so spatial and temporal patterns based morphospecies data may be confounded by cryptic and polymorphic species^12, 13^.

In recent years, culture-independent molecular techniques have been applied to study ciliates in a wide variety of marine environments^14–18^. Compared with other habitats, only three investigations using molecular methods, i.e. clone library, DGGE and high throughput sequencing (HTS), have been performed on estuarine ciliates^19–21^. Although these revealed higher taxonomic richness than microscopic surveys, they did not test^19^ or failed to find^20, 21^ relationships between ciliate community composition and environmental factors. Also, the studies were conducted at a handful of locations in the same sampling area, and did not consider the effect of temporal changes. HTS holds the potential to detect species even when present in low abundance and for sufficient representative taxa of the targeted area to be obtained, thus avoiding the bias associated with morphological and classic molecular methods. Furthermore, recent studies on prokaryotes have shown the importance of differentiating the active from the total communities^22–24^. This concept, however, has rarely been applied to ciliate communities^25^. Therefore, exploration of spatial and temporal patterns of distribution using rRNA-based HTS appears to be a promising approach for revealing the structure and stability of ciliate communities and for investigating relationships between community composition and environmental factors.

Abundant members of the biosphere attracted much attention in early ecological and biodiversity studies because of their ease of collection and likely influence on biogeochemical cycles. Recently, HTS techniques have broadened the scope of biodiversity, with the discovery of the “Rare Biosphere”. The rare biosphere, a term coined by ref. 26, is now defined as the group of taxa that found in a range of 0.01–1% of total abundance in microbial communities^27–30^. Several investigations have revealed that eukaryotic assemblages can rapidly and regularly reconstruct themselves and rare taxa may contribute to such shifts in community structure, becoming dominant when environmental conditions are favorable^31–33^. In a recent study, Logares *et al*. found that up to 30% of the sequences per sample belonged to neither rare nor abundant groups, but rather to an intermediate group^34, 35^. Despite its considerable relative abundance, this intermediate group is understudied compared with abundant and rare groups. In a study of short-term dynamics of lacustrine small eukaryotes, Mangot *et al*. found that freshwater microeukaryotes were largely composed of rare OTUs that never became abundant, and a core group that involved about 20 abundant OTUs and a handful of intermediate OTUs which sporadically transitioned into dominant taxa during the study period^36^. This study was conducted at a permanent station in Lake Geneva for one summer with relative stable environmental conditions^36^. Whether this rule can be applied to a more dynamic environment on a wider spatial and temporal scales is not known. Also, the metabolic activity/inactivity of the intermediate biosphere and the role it might play in the overall community remains largely unknown.

Here, we investigated the stability of community composition and structure of ciliates across the full salinity gradient in the Jiulong River Estuary near the southwestern Taiwan Strait in southeast China. A large dataset of 18 S rRNA and rDNA fragments were produced from 97 samples collected from one to three water depths at two-month intervals in 2014. The fine-scale spatial and temporal coverage, encompassing the full length of the estuary with replicate samples collected throughout the year, combined with ultra-deep sequencing for both 18 S rRNA and rDNA, enabled shifts in ciliate community composition and structure to be determined at high resolution across space and time. The present study aims to address the following questions: (i) Is the ciliate community composition and structure stable on a seasonal time-scales across the full salinity gradient? (ii) Are changes in community composition correlated with environmental factors? (iii) Considering their likely considerable relative abundance, are communities of intermediate forms stable or do they transit between being abundant and rare across space and time?

## Results

### Community composition

In rarefaction analysis, OTU richness approached saturation in most local communities (32–186 OTUs) and the total ciliate community (548 OTUs; Fig. S1). The reads derived from the total community were dominated by the class Spirotrichea (56% of relative abundance), followed by Oligohymenophorea (24.4%), Litostomatea (15.2%) and Prostomatea (2.3%), whereas the relative abundances of other groups, e.g. Phyllopharyngea, Heterotrichea and Colpodea, were each on average less than 1% (Fig. S2). The spatial distribution patterns of the two largest classes, Spirotrichea and Oligohymenophorea, showed opposite trends, with relative abundances of spirotricheans decreasing from euhaline, through polyhaline, mesohaline and oligohaline, to freshwater, while relative abundances of oligohymenophoreans increased with decreasing salinity (Fig. S2). Litostomateans and prostomateans both peaked in the mesohaline zone. At subclass rank Oligotrichia (39%), Choreotrichia (15.7%), Peritrichia (13.5%) and Haptoria (9.8%) accounted for the majority of read numbers.

### Beta-diversity and its shaping factors

**Figure 1:**
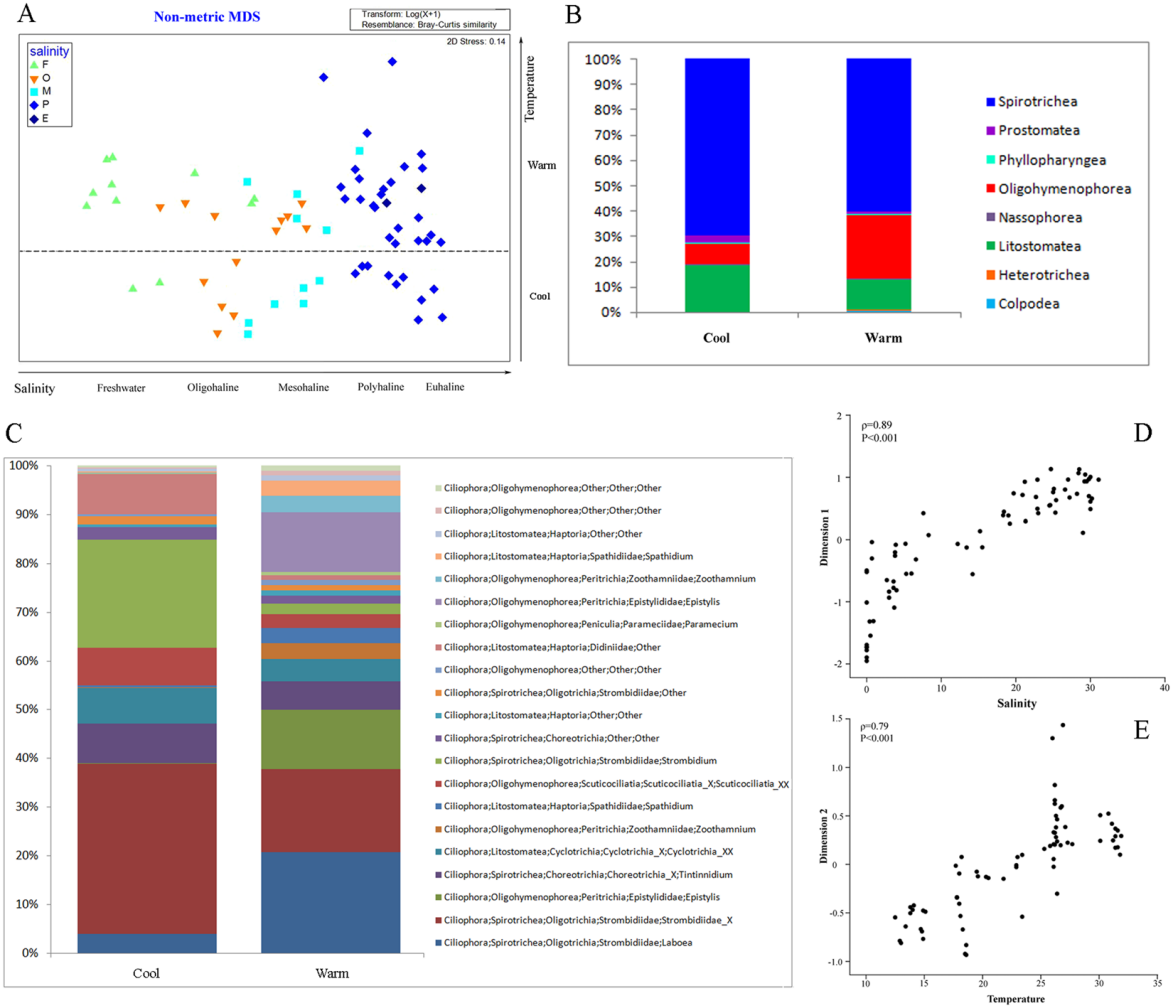
(**A**) Plot of non-metric multidimensional scaling based on Bray-Curtis distance matrix, showing pattern of beta diversity for all samples. Note that samples in the cool group were well confined to different salinity zones in contrast to the pattern seen in the warm group. (**B**,**C**) Assemblage compositions of the two seasonal groups at class and genus/species level, respectively. (**D**,**E**) Correlations of Dimension 1 with salinity, and Dimension 2 with temperature from Fig. 1a, respectively. Spearman’s rho values of 0.89 (P < 0.001) and 0.79 (P < 0.001) indicate strong relationships between salinity/temperature and community variation.

**Figure 2:**
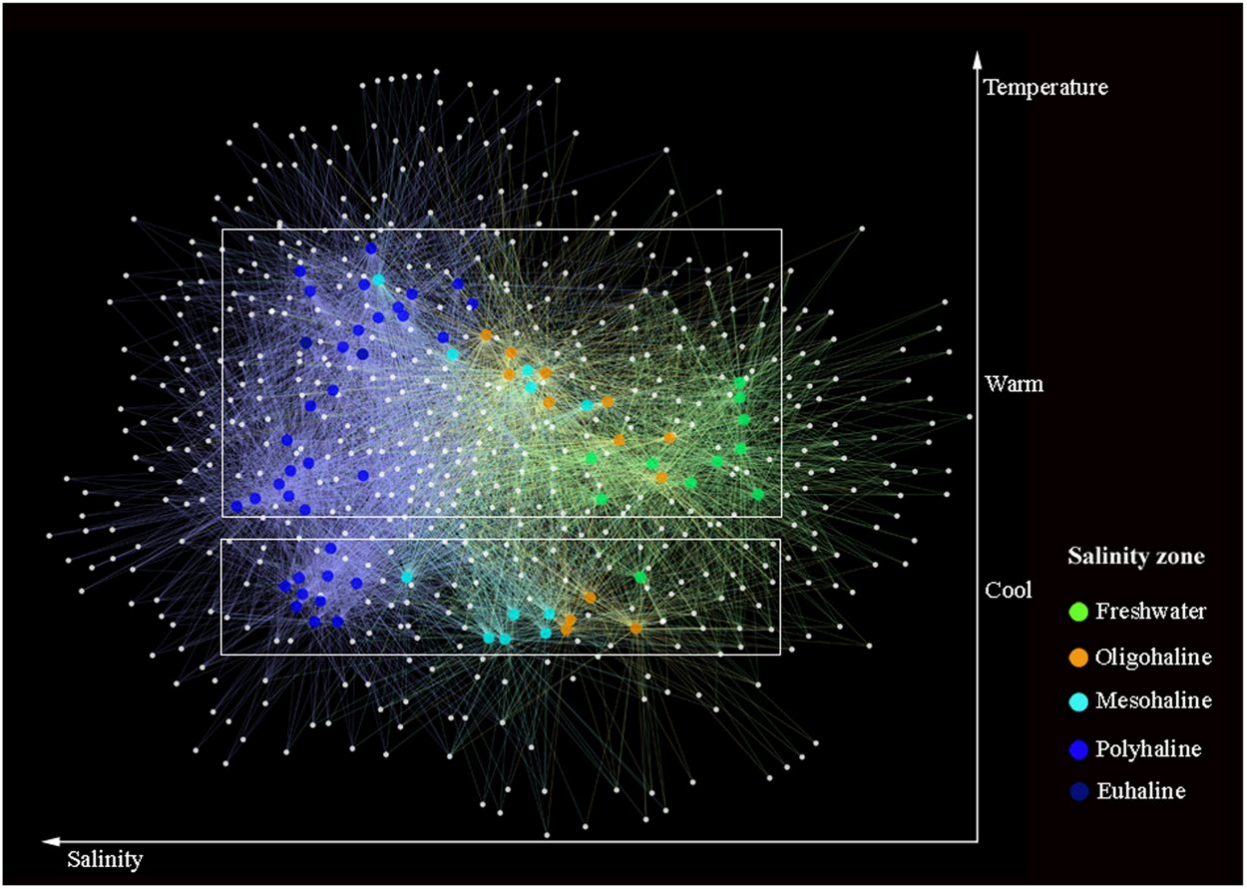
Operational taxonomic unit (OTU) network analysis of communities from all samples. Large nodes represent samples, whereas small nodes represent OTUs. The most relevant structuring features were salinity and temperature.

**Figure 3:**
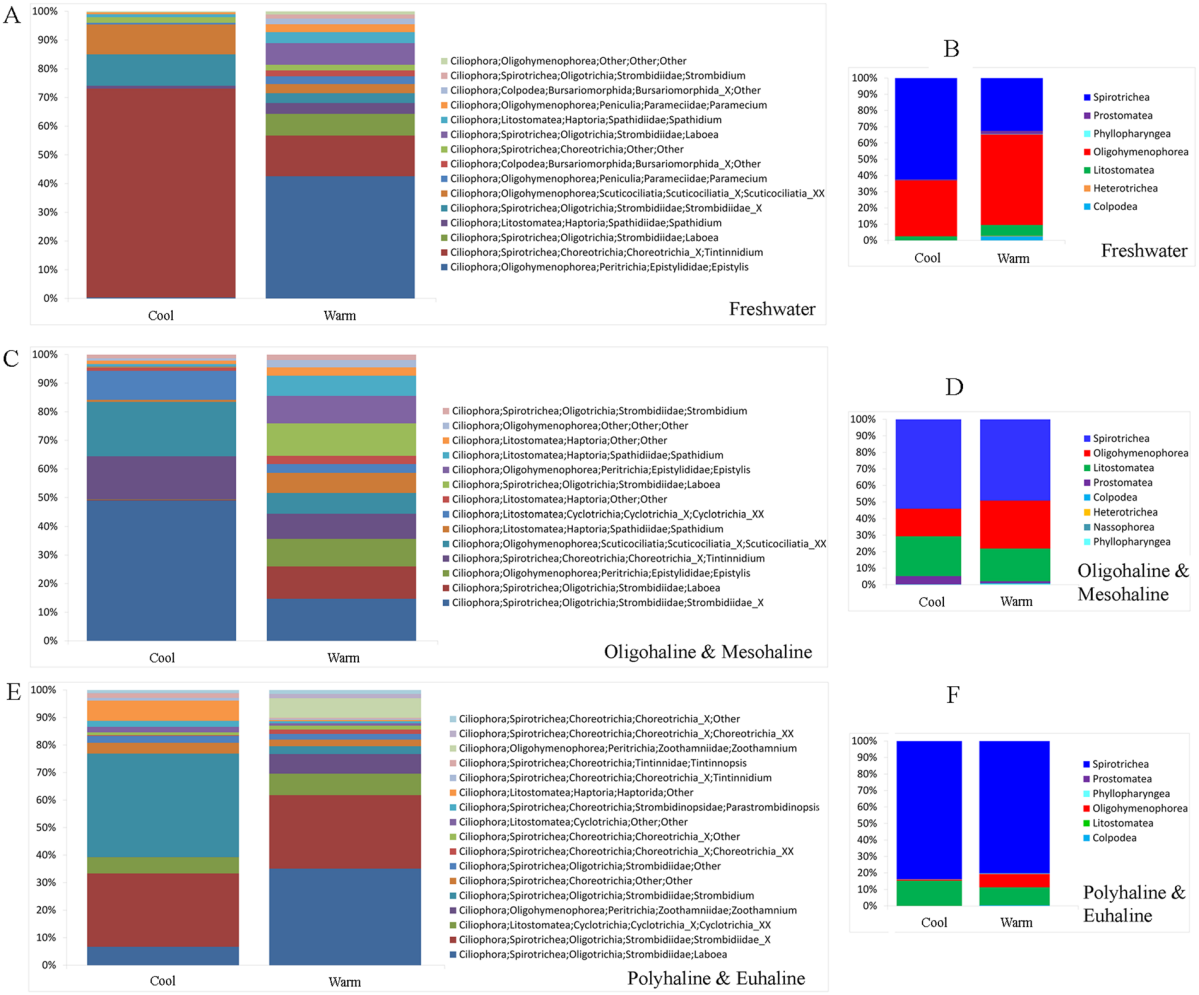
(**A**,**C**,**E**) Genus/species level compositions of the cool and warm subgroups within each salinity group. (**B**,**D**,**F**) Class-level compositions of the cool and warm subgroups within each salinity group.

Based on statistical analyses, the ciliate communities were divided into three distinct groups (ANOSIM, P < 0.001) corresponding with salinity zones in Jiulong River Estuary (JRE): freshwater (F), oligohaline-mesohaline (OM), and polyhaline-euhaline (PE) groups. The F group included samples with salinity less than 0.5‰, the PE group consisted of samples with salinity range of 18–40‰, and the OM group from samples with salinity between the F and PE groups (0.5–18‰). Similarities among samples were higher within groups than between groups (Table S3). The non-metric multidimensional scaling (nMDS) diagram of all samples revealed five groups separated along the two axes, comprising F, OM and PE groups along the first axis and cool and warm groups along the second axis (Fig. 1A). The first axis was clearly related to salinity since there was a strong correlation between dimension 1 and salinity (ρ = 0.89, P < 0.001, Fig. 1D). The second axis is related to temperature although the correlation between dimension 2 and temperature was weaker (ρ = 0.79, P < 0.001, Fig. 1E). Therefore, the ciliate community showed a clear spatial and temporal distribution pattern throughout the estuary. The ciliate communities shifted along a seasonal continuum from February to December, and could be separated into two distinct groups: cool (February, April, and December) and warm (June, August, and October) (ANOSIM, P < 0.001). For the total community and the three salinity groups, seasonal patterns were evident in both the nMDS and OTU-network diagrams (Figs 1A and 2). Although cool and warm groups were similar in phylogenetic composition at class level (Fig. 1B), the taxonomic identity at genus/species level and relative abundance of the core members of the two groups were dramatically different (Fig. 1C and Table 1). Within each of the three salinity groups, unique fingerprints of taxonomic composition consisting of different percentage of dominant OTUs displayed in cool and warm groups (Fig. 3). It was evident that one to a few OTUs dominated the cool and warm groups within each salinity group (Fig. 3A,C and E). In freshwater, a tintinnid *Tintinnidium* OTU accounted for 44.7% of the cool community, whereas a peritrich *Epistylis* OTU dominated the warm community (Fig. 3A). In oligohaline and mesohaline zones, an oligotrich Strombidiidae OTU accounted for 35.3% of the cool group, whereas four OTUs of *Laboea*, *Epistylis*, *Tintinnidium*, and Strombidiidae accounted for over 50% of the warm group (Fig. 3C). In polyhaline and euhaline community, an oligotrich *Strombidium* OTU made up 32.9% of the cool group, while an oligotrich *Laboea* OTU dominated the warm group (Fig. 3E). Contrary to the total community (ANOSIM, R = 0.469, P < 0.001) and the cool group (ANOSIM, R = 0.774, P < 0.001), hypothesis testing showed that community compositions of the warm group between mesohaline and polyhaline zones were indistinguishable (ANOSIM, R = 0.186, P > 0.05). This is also seen in nMDS and OTU network diagrams where mesohaline and polyhaline communities overlap, indicating only small spatial differences between them (Figs 1A and 2). In contrast to salinity and temperature, community compositions at different depths, i.e. surface, middle, and bottom layers, did not show statistically significant differences (Table S4).

**Table 1:** OTUs typically associated with abundant (>1%) OTUs in cool and warm groups, respectively.

Cool group			Warm group		
OTU name	Status in warm group	Taxonomy	OTU name	Status in cool group	Taxonomy
OTU_11 (5.89)	I	Spirotrichea, Oligotrichia, Strombidiidae, *Strombidium*, *Strombidium basimorphum*	OTU_4 (243.89)	I	Spirotrichea, Oligotrichia, Strombidiidae, *Laboea*, *Laboea strobila*
OTU_239 (12.48)	I	Spirotrichea, Oligotrichia, Strombidiidae, *Strombidium*	OTU_146 (1994.39)	R	Spirotrichea, Oligotrichia, Strombidiidae, *Laboea*, *Laboea* sp
OTU_5 (7.41)	I	Spirotrichea, Oligotrichia, Strombidiidae, *Sinistrostrombidium*	OTU_629 (3350.59)	R	Spirotrichea, Oligotrichia, Strombidiidae, *Laboea*, *Laboea* sp
OTU_12 (5.00)	I	Spirotrichea, Choreotrichia, Tintinnida,	OTU_528 (5.12)	I	Spirotrichea, Oligotrichia, Strombidiidae
OTU_21 (179.84)	R	Spirotrichea, Choreotrichia, Tintinnida	OTU_240 (3.77)	I	Spirotrichea, Oligotrichia, Strombidiidae
OTU_19 (56.03)	I	Spirotrichea, Choreotrichia, Choreotrichida, Strombidinopsidae, *Strombidinopsis*	OTU_705 (3.26)	I	Spirotrichea, Oligotrichia, Strombidiidae
OTU_16 (126.13)	I	Spirotrichea, Choreotrichia, Choreotrichida, Strombidinopsidae	OTU_14 (2268.29)	R	Spirotrichea, Choreotrichia, Tintinnida, Tintinnidae, *Tintinnidium*, *Tintinnidium* sp
OTU_37 (3146.75)	R	Spirotrichea, Choreotrichia, Choreotrichida, Strobilidiidae, *Rimostrombidium*	OTU_15 (4.70)	I	Spirotrichea, Choreotrichia, Tintinnida, Tintinnidae, *Tintinnidium*, *Tintinnidium* sp
OTU_41 (8.25)	I	Litostomatea, Haptoria, Haptorida, Didiniidae, *Cyclotrichium*	OTU_13 (15645.37)	R	Oligohymenophorea, Peritrichia, Zoothamniidae, *Zoothamnium*
OTU_8 (10.15)	I	Litostomatea, Haptoria, Haptorida, Didiniidae	OTU_3 (520.20)	I	Oligohymenophorea, Peritrichia, Epistylididae, *Epistylis*, *Epistylis* sp
OTU_17 (709.47)	R	Litostomatea, Haptoria, Haptorida, Didiniidae,	OTU_22 (118.65)	I	Litostomatea, Haptoria, Haptorida, Actinobolinidae
OTU_28 (3.48)	I	Litostomatea, Haptoria, Haptorida, Didiniidae	OTU_661 (3.98)	I	Litostomatea,Haptoria, Haptorida, Didiniidae
OTU_24 (3.47)	I	Litostomatea, Haptoria, Cyclotrichida, Mesodiniidae, *Askenasia*	OTU_9 (6.39)	I	Litostomatea, Haptoria, Cyclotrichida
OTU_20 (272.24)	R	Prostomatea, Prorodontida, Urotrichidae, *Urotricha*	OTU_33 (8.01)	I	Litostomatea,Haptoria, Cyclotrichida

Numbers in brackets indicate the ratio of relative abundance of each OTU in one group to its relative abundance in the other group. Abbreviation: I, intermediate; R, rare.

**Figure 4:**
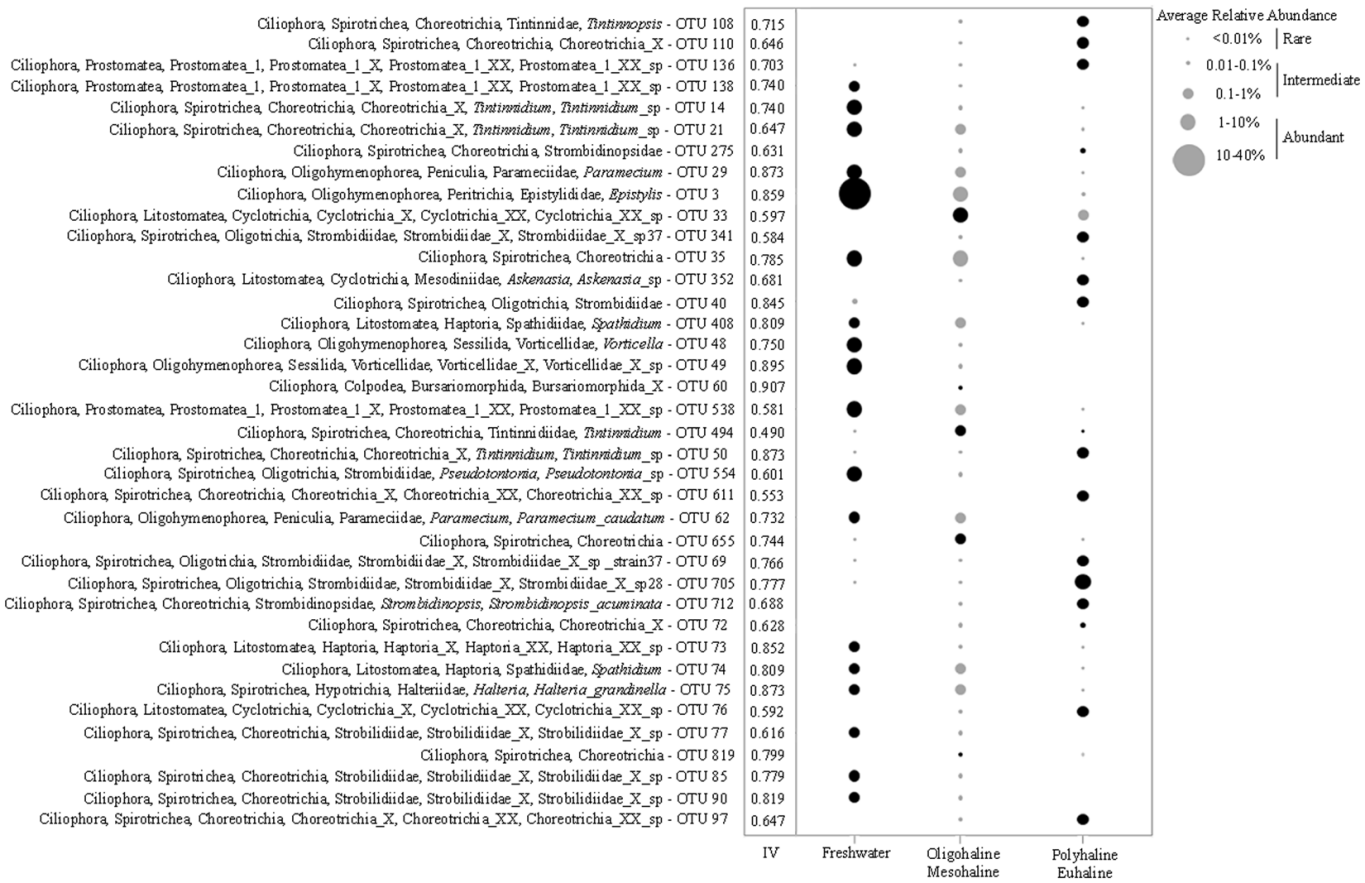
Bubble plot of the top indicator OTUs in each salinity zone. The size of the bubble indicates the average relative abundance (%) of each OTU in each of the three salinity environments. Black shaded bubbles show the salinity zone for which OTU are indicators. IV: Indicator Value.

Comparison of all samples to the environmental dataset showed that salinity, temperature, Chl *a*, and violaxanthin explained most of the community variability across the entire community dataset, with a rank coefficient of ρ = 0.669 (Table 2). Among the biotic- and abiotic factors, salinity was the most important variable with a rank coefficient of ρ = 0.615 (Table 2). In each of the three salinity groups, community compositions varied seasonally and correlated with water temperature, with ρ values ranging from 0.404 to 0.789 (Table 2). In freshwater, the inclusion of Chl *a*, NH_4_, DRP and diadinoxanthin increased the correlation to ρ = 0.807. The top indicator species for freshwater was a peritrich *Epistylis* OTU with an indicator value (IV) of 0.859, which accounted for 40% of the freshwater group (Fig. 4). The oligohaline and mesohaline communities varied seasonally, and correlated with temperature (ρ = 0.404) and diadinoxanthon (ρ = 0.219; Table 2). The variables were elevated in warm group (summer and autumn) compared with those in cool group (spring and winter) with average temperature increasing from 16.5 to 26.4◦C and average diadinoxanthin concentration from 0.41 to 0.59 μg/L. Indicator analysis showed the top indicator for the oligohaline and mesohaline zones was a cylotrichiid OTU (IV: 0.597) (Fig. 4). In the polyhaline and euhaline zones, temperature (ρ = 0.561) and violaxanthin (ρ = 0.367) were most important factors influencing community variability (Table 2). In the polyhaline and euhaline zones the top indicator was a Strombidiidae OTU (IV: 0.777) (Fig. 4). The environmental variables that strongly correlated with the salinity zone-specific OTUs in each salinity groups were generally the same variables that correlated with overall community variability with differing degrees (Table 2). Simple- and Partial Mantel tests showed that cool and warm groups responded to environmental factors in opposite ways, e.g. cool group showed correlations with temperature, bacteria, and zeaxanthin and was uncorrelated with chlorophyll c2, chlorophyllide a, fucoxanthin, violaxanthin, lutein, and chlorophyll *b*, whereas the warm group showed the reverse (Table 3).

**Table 2:** BVSTEP and BIOENV analyses showing the correlations between community compositions and environmental variables.

Environment	BV-STEP factors	ρ	BIO-ENV factors	ρ	Variability explained
Environments with all OTUs					
All	Salinity, Temperature, Chl *a*, Violaxanthin	0.669	Salinity	0.615	24.52%
			Violaxanthin	0.408	
Freshwater	Temperature, Ch l *a*, NH_4_, DRP, Diadinoxanthin	0.807	Temperature	0.798	71.18%
			DRP	0.702	
Oligohaline & Mesohaline	Salinity, Temperature, Chl *a*, DRP, Chl *b*, Nanophytoplankton ratio	0.388	Temperature	0.404	42.74%
			Diadinoxanthin	0.219	
Polyhaline & Euhaline	Salinity, Temperature, DRP, Violaxanthin, Chl *b*	0.576	Temperature	0.561	33.03%
			Violaxanthin	0.367	
Environments with only indicator OTUs					
Freshwater	Temperature, Chl *a*, DRP, Chl *b*	0.838	DRP	0.806	69.91%
			Temperature	0.783	
Oligohaline & Mesohaline	Temperature, Bacteria, NH4, Diadinoxanthin	0.494	Temperature	0.528	46.96%
			Diadinoxanthin	0.325	
Polyhaline &Euhaline	Temperature, DRP, Microphytoplankton ratio	0.664	Temperature	0.563	34.35%
			Zeaxanthin	0.479

**Table 3:** Simple and partial Mantel tests for the correlations between environmental factors and community variability.

	Simple Mantel Test						Control for	Partial Mantel Test					
	Total		Cool		Warm			Total		Cool		Warm	
	r	P	r	P	r	P		r	P	r	P	r	P
Environmental factors													
Salinity	0.658	**0.0001** ^*^	0.781	**0.0001** ^*^	0.607	**0.0001** ^*^	Temperature	0.521	**0.0001** ^*^	0.514	**0.0001** ^*^	0.347	**0.0001** ^*^
							Chl *a*	0.644	**0.0001** ^*^	0.638	**0.0001** ^*^	0.554	**0.0001** ^*^
							Bacteria	0.633	**0.0001** ^*^	0.580	**0.0001** ^*^	0.511	**0.0001** ^*^
Temperature	0.189	**0.0001** ^*^	0.734	**0.0001** ^*^	0.531	**0.0001** ^*^	Salinity	0.127	**0.0100** ^*^	0.362	**0.0004** ^*^	−0.001	0.4945
Depth	−0.009	0.5306	−0.024	0.5509	−0.025	0.6250	Salinity	−0.045	0.7932	−0.105	0.9005	−0.038	0.7132
Chl *a*	0.259	**0.0001** ^*^	0.602	**0.0001** ^*^	0.314	**0.0001** ^*^	Salinity	0.193	**0.0008** ^*^	0.172	**0.0137**	0.102	**0.0399**
Bacteria	0.259	**0.0003** ^*^	0.650	**0.0001** ^*^	0.386	**0.0001** ^*^	Salinity	0.118	**0.0140**	0.138	**0.0372**	−0.066	0.9268
Accessory pigments													
Chlorophyllide a	0.393	**0.0001** ^*^	0.530	**0.0001** ^*^	0.447	**0.0001** ^*^	Salinity	0.142	**0.0028** ^*^	0.082	0.1623	0.198	**0.0017** ^*^
Chlorophyll c2	0.215	**0.0001** ^*^	0.344	**0.0002** ^*^	0.241	**0.0003** ^*^	Salinity	0.134	**0.0046** ^*^	0.053	0.2372	0.195	**0.0027** ^*^
Fucoxanthin	0.280	**0.0001** ^*^	0.466	**0.0001** ^*^	0.317	**0.0001** ^*^	Salinity	0.174	**0.0005** ^*^	0.067	0.1249	0.232	**0.0010** ^*^
Neoxanthin	0.422	**0.0001** ^*^	0.372	**0.0002** ^*^	0.551	**0.0001** ^*^	Salinity	0.171	**0.0008** ^*^	0.224	**0.0064** ^*^	0.342	**0.0001** ^*^
Violaxathin	0.477	**0.0001** ^*^	0.528	**0.0001** ^*^	0.456	**0.0001** ^*^	Salinity	0.310	**0.0001** ^*^	0.078	0.1551	0.371	**0.0001** ^*^
Diadinoxanthin	0.331	**0.0001** ^*^	0.340	**0.0003** ^*^	0.376	**0.0002** ^*^	Salinity	0.168	**0.0014** ^*^	0.170	**0.0188**	0.244	**0.0004** ^*^
Alloxathin	0.403	**0.0001** ^*^	0.524	**0.0001** ^*^	0.415	**0.0001** ^*^	Salinity	0.170	**0.0005** ^*^	0.052	0.2413	0.138	**0.0173**
Zeaxanthin	0.387	**0.0001** ^*^	0.665	**0.0001** ^*^	0.284	**0.0001** ^*^	Salinity	0.043	0.1430	0.200	**0.0191**	0.084	0.0583
Lutein	0.578	**0.0001** ^*^	0.687	**0.0001** ^*^	0.597	**0.0001** ^*^	Salinity	0.164	**0.0005** ^*^	0.128	0.0748	0.261	**0.0004** ^*^
Chlorophyll *b*	0.481	**0.0001** ^*^	0.621	**0.0001** ^*^	0.440	**0.0001** ^*^	Salinity	0.227	**0.0002** ^*^	0.025	0.3422	0.282	**0.0003** ^*^
Phytoplankton ratio													
Microphytoplankton ratio	0.160	**0.0004** ^*^	0.217	**0.0109**	0.122	**0.0204**	Salinity	0.137	**0.0013** ^*^	0.081	0.1672	0.117	**0.0255**
Nanophytoplankton ratio	0.0277	0.2580	0.1026	0.1220	0.0088	**0.0004** ^*^	Salinity	−0.023	0.6690	−0.007	0.4873	−0.021	0.6205
Picophytoplankton ratio	0.1857	**0.0009** ^*^	0.3002	**0.0027**	0.1486	**0.0125**	Salinity	−0.056	0.8780	−0.010	0.4983	0.014	0.3756

Only top abundant accessory pigments were included in the analysis. Contrasting response pattern between cool and warm groups were shaded in grey. Definitions of ratios used as proxies for phytoplankton functional groups followed Vidussi *et al*. (2001) and Uitz *et al*. (2006). ^*^ indicates P value is still significant after Bonferroni correction.

### Pattern of intermediate biosphere

**Figure 5:**
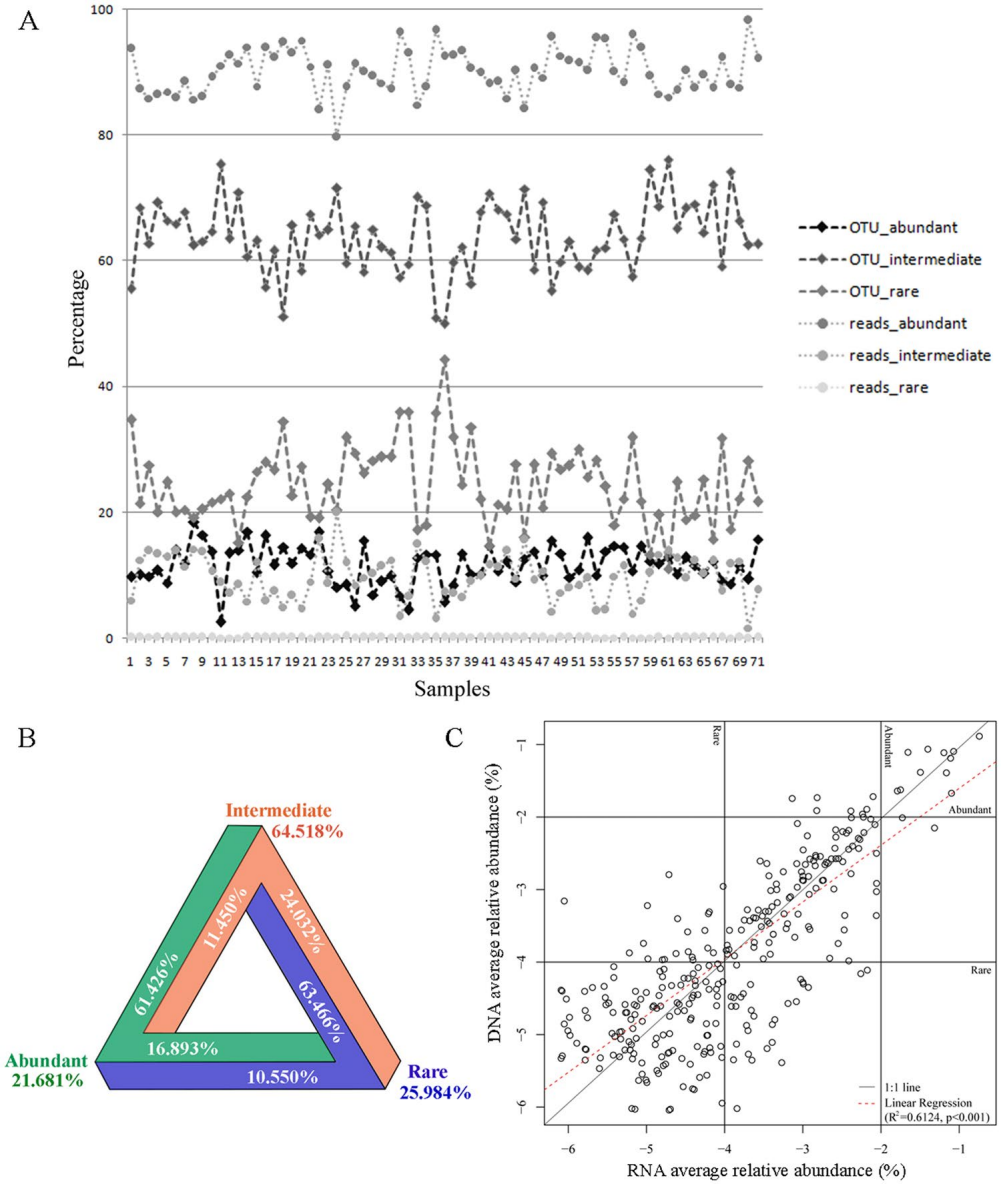
(**A**) Percentage of the abundant, intermediate, and rare reads and OTUs across all samples. (**B**) Percentage of the abundant, intermediate, and rare OTUs in transition among the three biospheres across all samples. For example, to test whether the intermediate group was stable or shifted between the abundant and rare groups across all samples, the numbers of OTUs belonging to the abundant, intermediate and rare groups in the intermediate OTU table were counted separately and then divided by the total number of OTUs to calculate the proportion of each group represented in the intermediate biosphere. (**C**) Average relative abundance of common OTUs occurring in both rRNA and rDNA datasets. The abundance thresholds for abundant (>1%) and rare (<0.01%) are indicated with horizontal and vertical lines. The best-fitting linear regression which was a relatively Y-axis deviated 1:1 relationship.

To explore patterns of distribution of the intermediate biosphere, the abundant, intermediate, and rare communities of total samples were analyzed. The proportions of the abundant, intermediate, and rare OTUs were relatively constant across all samples collected, with ranges of 2.6–18.4% for the abundant OTUs, 50.0–76.1% for the intermediate OTUs and 11.0–44.3% for the rare OTUs (Fig. 5A). Reads corresponding to the abundant OTUs represented on average 90.2% (SD = 3.6) of all samples, whereas those of the rare OTUs represented on average 0.16% (SD = 0.05) (Fig. 5A; Table S5). In all samples investigated, the proportion of reads per sample belonged to the intermediate group ranged from 1.65% to 20.0% with an average of 9.73% (Table S5). The intermediate group possessed the highest OTU richness accounting for 94.53% of the total community, followed by the rare (83.39%) and abundant (27.19%) groups. To address whether the intermediate group was stable or cycled between the abundant and rare groups, the transitions among the three groups were explored for all samples. The results revealed that all three groups were dynamic and that the intermediate group had the highest proportion of taxa which transitioned into another group, i.e. abundant or rare. For the intermediate group, about 65% remained in the intermediate group while about 24% and 11% transitioned into rare and abundant groups, respectively (Fig. 5B). For the rare group, about 26% remained in that group, and about 63% and 11% transitioned into intermediate and abundant groups, respectively. Among the abundant OTUs, about 22% remained in the abundant group, with 61% and 17% transitioning into the intermediate and rare groups, respectively (Fig. 5B).

In order to examine the metabolic activity of the intermediate group, 26 samples were collected in February and June from the estuary, from which both 18 S rRNA and rDNA sequences were obtained. The relationship between 18 S rRNA and rDNA frequency of common OTUs that were present in both RNA and DNA datasets was then examined. The relative abundance of common OTUs showed a deviation from a 1:1 relationship with the Y-axis, indicating that most OTUs of the intermediate and abundant groups likely had higher metabolic activity rates compared to the average whereas the rare group had relatively low metabolic activity (Fig. 5C).

## Discussion

Previous studies on the dynamics of estuarine ciliate communities were restricted to one or two dimensions, being based either on horizontal surveys across salinity gradients, time series at one to two fixed locations, or spatial and temporal variations of a single assemblage of ciliates^8, 20, 21, 37, 38^. Here we compare the community composition of full spectrum of estuarine ciliates in three dimensions: horizontally, from freshwater to the euhaline zone; vertically, from surface to bottom waters, and; temporally, over a 12-month period. This three dimensional study, combined with ultra-deep sequencing for both 18 S rRNA and rDNA, enabled shifts in community compositions and structures to be determined at high resolution. The results revealed that the spatial distribution pattern of ciliate communities was divided into three distinct groups, i.e. freshwater, oligohaline-mesohaline, and polyhaline-euhaline. Spatial differentiation among communities was highly correlated with salinity, confirming the findings of a 2-year study of ciliate community dynamics in a eutrophic estuary (Bay of Biscay) based on morphological characters^10^. This study demonstrated that the ciliate communities were delineated into three main groups that were composed of taxa mainly appearing in freshwater, mid-estuary, and seaward zones, respectively.

In contrast to the horizontal variation, ciliate community variability did not show a correlation with depth (Tables 2, 3 and S4). This might due to the estuarine waters being well mixed, thereby supporting similar communities in the surface, middle, and bottom layers and giving a weak depth signal. Tamura *et al*. also found that ciliate communities in river and plume surface waters resembled that in the deep river water in Long Island Sound and suggested physically vertical mixing might be one of the reasons for this^21^.

In an estuary, salinity contributes to density gradients that physically separate water masses and the microbial communities that reside within them^39^. However, the degree to which water masses are mixed/separated largely depends on the magnitude of mixing caused by river flow and tides. This mixing of waters from surface to bottom layers and from euhaline to freshwaters can lead to the formation of ciliate communities in zones that comprise populations from multiple water mass sources. Our results support this hypothesis in that oligohaline-mesohaline group (OM) included OTUs from the freshwater (F) and polyhaline-euhaline groups (PE), sharing 43.8% and 56.6% of OTUs with F and PE, respectively (Fig. S3). This indicated that community composition of estuarine ciliates was influenced more by coastal ocean than river flow. However, there was still 7% of OTUs that was OM specific (Fig. S3). Examining indicators of the OM group also revealed that the majority of abundant indicator OTUs were either absent or appeared as intermediate/rare forms in the F and PE groups. Statistical analyses showed that the community composition of the OM group differed significantly from that of the F and PE groups (ANOSIM, P < 0.001), suggesting that the formation of a distinct estuarine community (OM group) was probably due to the doubling time of ciliate populations exceeding the rate at which cells are lost due to flushing by water flow.

Temporal variability in the ciliate community was evident, forming cool and warm groups in the total community and within all three salinity groups (Figs 1A and 3). Several studies of estuarine ciliates identified temperature as the principle cause of community variability^11, 40^. A study on tintinnid ciliates in Kaštela Bay (middle Adriatic Sea) revealed that species distribution was strongly affected by temperature, which was the most important environmental factor determining their seasonality^40^. Mironova *et al*. identified a similar pattern among 30 surface water samples collected from two sites in Neva Estuary (Baltic Sea) in which two distinct groups succeeded each other during different seasons^11^. In the present study, community composition in the warm assemblages in the mesohaline and polyhaline zones were indistinguishable (ANOSIM, R = 0.186, P > 0.05; Figs 1A and 2). This contrasted with the cool assemblages which showed distinct differences in these two salinity zones. Possible explanations for these findings include: (1) rates of dispersal from the mesohaline to the polyhaline zone were higher during the warm seasons, or; (2) ciliate growth rates in warm waters were sufficiently high to overcome the physical processes that would otherwise flush them out of the system in cool waters.

Besides salinity and temperature, ciliate community variability was also correlated with chl *a* (Table 2). Furthermore, Simple- and Partial Mantel tests showed significant correlations with bacteria, accessory pigments and microphytoplankton ratio, suggesting ciliate communities in JRE were not only shaped by abiotic factors, i.e. salinity and temperature, but also controlled by biotic factors, e.g. bacteria and micophytoplankton (Table 3). Because of the differential response of cool and warm groups to salinity and temperature, correlations between the two groups and other environmental variables were investigated. This indicated a contrasting response of the two groups to prey abundance proxies (Table 3). Therefore, combined with the dramatic differences in their taxonomic identity (Fig. 1C), differences in the relative abundance of core members (Table 1), and their differential responses to environmental factors (Table 3), the present study revealed that species belonging to cool and warm groups occupied different ecological niches, therefore suggesting seasonal niche separation of ciliates in JRE.

No previous study has addressed both temporal and spatial variability of estuarine ciliates employing HTS yet, which will definitely underestimate the contribution of less abundant members of the community, i.e. the intermediate and rare groups. Two pioneering studies on spatial distribution patterns of ciliates across salinity gradients using molecular methods, i.e. DGGE and clone library, were performed in Long Island Sound based on 12 and 10 samples, respectively^20, 21^. Neither study recovered a clear relationship between community variability and environmental parameters. Possible causes for this include the limited sampling or the low sequencing depth. In order to address the possible causes, the total community in the present study was randomly resampled to 100 sequences per sample corresponding to the general sequencing effort of clone library. The variability of the total community/the bimonthly collected communities was re-compared to environmental variables to determine whether or not the original correlation relationship was stable. The results revealed that even employing a restricted number of samples or samples with limited sequencing depth, it was still possible to identify environmental factors that explain community variability (Table S6). These findings suggest that the reason why community variability in the two previous studies could not be readily attributable to environmental factors was not related to undersampling or lack of sequencing depth, rather it might be due to more complex factors that were unexplored and/or were specific for that precise location^20, 21^.

Previous studies of prokaryotes and protists from a variety of environments have shed light on the structure of the abundant and rare biospheres, but left the group that is neither abundant nor rare (the ‘intermediate’ biosphere) largely ignored^22–24, 27–30, 34, 35, 41–43^. In this study, we showed that the intermediate group possessed the highest OTU richness across salinity gradients over the 1-year period, which is in accordance with the pioneering study of intermediate group focusing on lacustrine small eukaryotes^36^. Besides having the highest OTU richness, the intermediate group also had the highest proportion (65%) of transitions among the three groups (Fig. 5B). Indeed, when examining OTUs typically associated with abundant OTUs in cool and warm groups, 10 of 14 abundant OTUs in the former came from the intermediate group within the latter, and vice versa (Table 1). Also, analyses of the three salinity groups, i.e. F, OM, and PE, revealed that the majority of indicator OTUs in each belonged to the intermediate group (Fig. 4), suggesting a critical role for the intermediate group in community reassembly across spatial and temporal gradients.

The study by Mangot *et al*. was performed at a single location in Lake Geneva representing a relatively stable environment^36^. Nevertheless, both this and the present study showed that the intermediate groups make a contribution to community variability. Compared with the previous report, however, the present study revealed a higher contribution to the community variability, suggesting the important role of the intermediate group in maintaining community stability in a more dynamic environment. Analysis of the DNA:RNA ratio indicated that the intermediate forms are metabolically active because the regression line deviated from 1:1 line toward RNA axis (Fig. 5C). All this suggests that the intermediate group is a key sub-community in JRE and likely plays a key role in maintaining stability (as seen in showing the highest taxonomic richness and the highest proportion in transitions) and function (as seen in keeping metabolic activity) of the ciliate community in this highly dynamic environment across space and time.

The abundant, intermediate, and rare groups possessed relatively stable proportions of OTU richness over the 1-year period (Fig. 5A). Therefore, estuarine ciliate community can be considered to be constituted of three groups, i.e. the abundant, intermediate, and rare, which presented not only regular proportions in relative abundance (considering 1% and 0.01% as grouping boundaries) but also in relative OTU richness. These findings are consistent with a previous study that explored patterns of abundant and rare microeukaryotes in six separate coastal locations in Europe^34^.

## Conclusions

This three-dimensional study revealed that spatial variation, seasonality and biological gradients defined the community composition of microzooplankton in the estuary, with spatial variability exceeding seasonal variability. Therefore, the present study shed light on how microzooplankton shifted with abiotic gradients and food resources that was essential for prediction of community dynamics. Furthermore, analyses of the abundant, intermediate, and rare biospheres revealed that the intermediate group possessed the highest OTU richness, highest proportion of taxa transiting between groups, high metabolic activity and contributed significantly to both spatial and temporal variability of the community. A pivotal role for this group in maintaining stability and function of microzooplankton communities in the highly dynamic estuarine environment is therefore posited. Overall, this study provides a better understanding of stability of community composition and structure of microzooplankton in a highly dynamic environment across both space and time, and offers evidences for the unique biogeographic pattern of microzooplankton communities at an entire scale of this estuary ecosystem.

## Methods

### Study site and sampling

The Jiulong River Estuary (JRE) is located in a subtropical monsoon zone of southeastern China, near southwestern end of the Taiwan Strait (Fig. S4). The estuary is shallow, with water depth ranging from 3 to 16 m. The total length and catchment area of JRE is 21 km and 100 km^2^, respectively. JRE is under the influence of semidiurnal tidal cycles, with the tidal range of 2.7–4 m from the upper to the lower estuary^44^. Sampling was carried out bimonthly over a calendar year (2014). A total of 97 samples were collected from eight sampling sites across the full salinity gradient from freshwater to euhaline (Fig. S4; Table S1). Site A5 was subject to the influence of the freshwater plume, while sites JY1, JY2, KM2 were influenced by coastal waters.

Surface waters were collected with Niskin Bottles. For freshwater site A5 and coastal site JY2, water was collected at three depths (surface, middle and bottom). For each sample, 2 L of water was pre-filtered with a 200 µm nylon mesh (Sefar Nitex) to remove mesozooplankton and then directly filtered onto a polycarbonate filter, 3 µm pore size (Millipore, USA) which was stored in RNAlater (Qiagen, Germany) at −20 °C. Water temperature, salinity, and depth were determined using a SeaBird CTD profiler. 1.8 ml of Water for bacterial analysis were pre-filtered with 20 µm mesh, fixed with ice-cool glutaraldehyde at a final concentration of 1%, stored in liquid nitrogen and later analyzed using a flow cytometer (Beckman Coulter, Epics Altra II)^45^. For chlorophyll a and pigment analyses, 200 ml of water was immediately filtered through 47 mm GF/F filter, stored in liquid nitrogen, and later extracted in acetone. Extracts were analyzed for both chlorophyll *a* and pigment composition by Trilogy fluorometer and Agilent series 1100 HPLC, respectively^46^. Water samples were also analyzed for nutrients including nitrate (NO_3_-N), nitrite (NO_2_-N), ammonia (NH_4_-N), dissolved total nitrogen (DTN) and dissolved reactive phosphorus (DRP) as described previously^47^. Information of environmental characteristics of the sampling sites is included in Table S2.

### High throughput sequencing and data analysis

High throughput sequencing of 97 samples were performed, with 71 samples from the whole year for RNA sequencing and 26 samples from both dry (February) and wet (June) seasons for DNA sequencing. All spatial and temporal distribution patterns were inferred from rRNA dataset. The rDNA dataset was used for revealing the metabolic activity of the intermediate group only. Extraction of DNA and RNA, reverse-transcription and PCR amplification followed^48^. Each sample was amplified in triplicate, pooled, and purified using the MiniElute Gel Extraction Kit (Qiagen, USA). All purified hypervariable V4 region amplicons were sent to Majorbio sequencing company (Shanghai, China) for sequencing using Illumina MiSeq platform. Short reads were deposited in NCBI Sequence Read Archive (accession code SRP115671).

Quality filtering, demultiplexing and assembly of raw data were performed with Trimmomatic^49^ and Flash software^50^. Chimeras were identified using UCHIME as implemented in USEARCH^51^. Operational Taxonomic Units (OTUs) clustering was done using Uparse at the 97% similarity cutoff ^52^. Generation of OTU tables and taxonomy assignment of each OTU were done with QIIME^53^ using the reference taxonomic database of ciliates from the Protist Ribosomal Reference database (PR2)^54^. Sequences were normalized by randomly resampling 14,825 reads per sample (the lowest number of sequences recovered for 97 samples), which minimized bias associated with sequencing depth and allowed for comparison of results for all samples.

### Abundant, intermediate, and rare biospheres

In addition to the total community, separate analyses of abundant, intermediate, and rare assemblages were performed. OTUs were considered to be abundant or rare when they comprised more than 1% or less than 0.01% of the reads in a sample, respectively^28, 29^. Between these two is the intermediate group^34, 36^. To test whether the intermediate group was stable or shifted between the abundant and rare groups across space and time, the proportion of the intermediate group in transition across all samples was calculated. In order to reveal metabolic activity of the intermediate group, linear regression of 18 S rDNA/rRNA ratio of samples collected in dry and wet seasons was performed in R.

### Statistical analysis

To assess similarities between ciliate communities, pairwise similarities among samples were calculated by Bray-Curtis similarity coefficient^55^. Ordination of samples was done by Non-Metric Multidimensional Scaling (nMDS) using Bray-Curtis dissimilarity matrices. OTU network was constructed in QIIME based on the normalized OTU table and visualized in Cytoscape^56^. Global and pairwise differences among groupings of samples were tested by ANOSIM within PRIMER V.6.0 package. To identify the specific OTUs that characterized each of the salinity zones, we used Indicator Species Analysis run in R using the package Indicspecies^57^. Only OTUs with indicator values (IV) > 0.3 and P < 0.05 were considered good indicators. Environmental data were compiled and variables not normally distributed were transformed as close to normality as possible. Analyses were completed with a reduced set of 65 samples due to missing environmental data in some samples. Spearman rank correlation coefficient (ρ) was used in BV-STEP and Bio-ENV to determine the degree of association between similarity matrices of 18 S rRNA amplicons and environmental data. Prior to performing the analyses, highly correlated environmental variables (ρ > 0.90) were removed to ensure more interpretable results. Partial Mantel tests were conducted in R with Vegan package to assess individual effects of salinity/temperature on β-diversity after controlling for salinity or temperature^55^.

## Electronic supplementary material

Supplementary file
