# Supplementary material for: Marked seasonality and high spatial variation in estuarine ciliates are driven by exchanges between the ‘abundant’ and ‘intermediate’ biospheres
Source: Sci Rep. 2017 Aug 25;7:9494. doi: 10.1038/s41598-017-10308-y (PMC5573402; doi:10.1038/s41598-017-10308-y)
Supplement: Supplementary file 1 — Supplementary file [file 41598_2017_10308_MOESM1_ESM.pdf]

Marked seasonality and high spatial variation in estuarine ciliates are driven by exchanges between the 'abundant' and 'intermediate' biospheres

Ping Sun[1, *], Liying Huang[1], Dapeng Xu[2], Bangqin Huang[1], Nengwang Chen[1] and Alan Warren[3]

[1]Key Laboratory of the Ministry of Education for Coastal and Wetland Ecosystem, College of the Environment and Ecology, Xiamen University, Xiamen 361102, China. [2]State Key Laboratory of Marine Environmental Science, Institute of Marine Microbes and Ecospheres, Xiamen University, Xiamen 361102, China. [3]Department of Life Sciences, Natural History Museum, London SW7 5BD, UK.

[*]Correspondence and requests for materials should be addressed to P.S. (psun@xmu.edu.cn)

Figure S1 Rarefaction curve for individual samples (left) as well as for the total community (right).

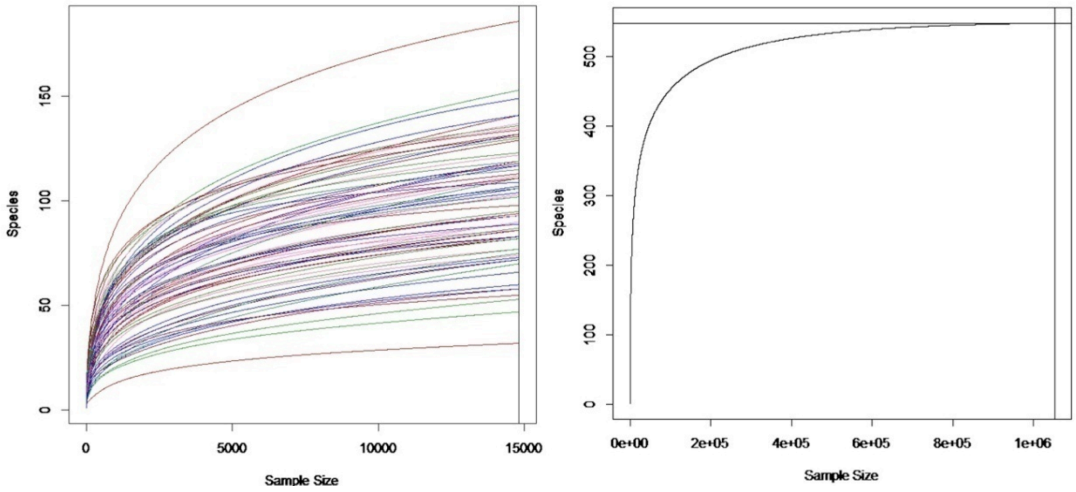

Figure S2 Variations of the taxonomic composition of the total community along salinity zones.

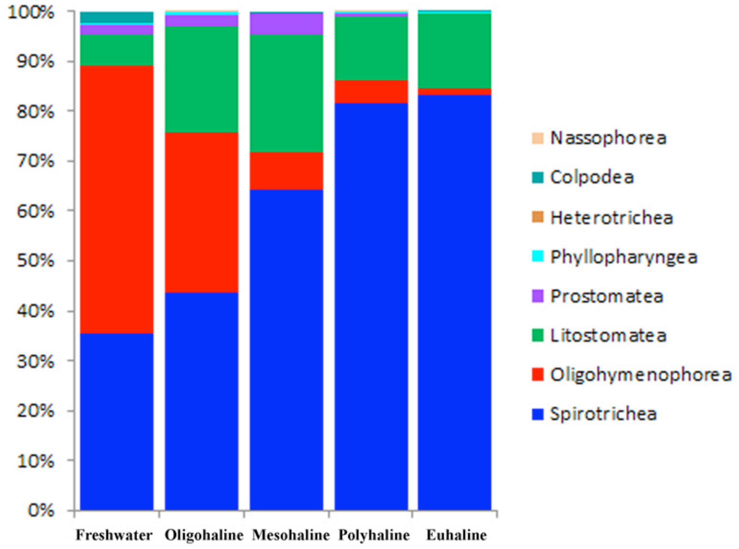

Figure S3 Venn diagram of OTUs from three delineated salinity groups: Freshwater, Oligohaline and Mesohaline, and Polyhaline and Euhaline.

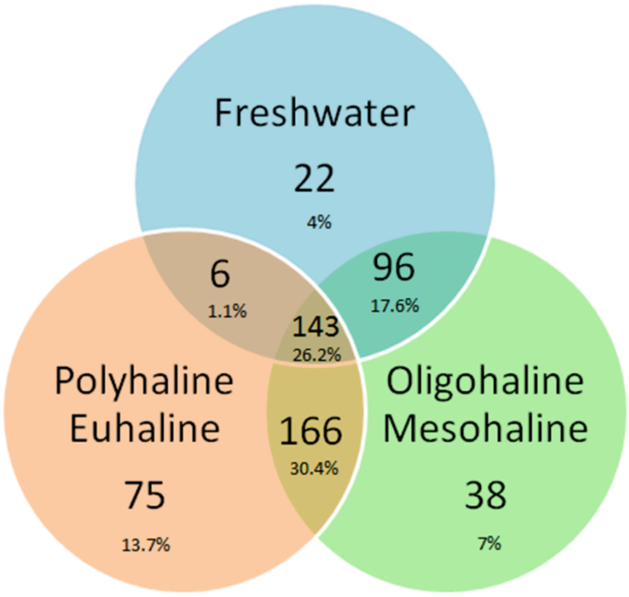

Figure S4 Location of sampling sites in Jiulong River Estuary. Inset depicts Fujian coast near southwestern Taiwan Strait. The sampling map was generated with Surfer version 7 (Golden Software, http://www.goldensoftware.com/products/surfer). The inset was generated with Ocean Data View version 4 (Schlitzer, R., Ocean Data View, odv.awi.de, 2017).

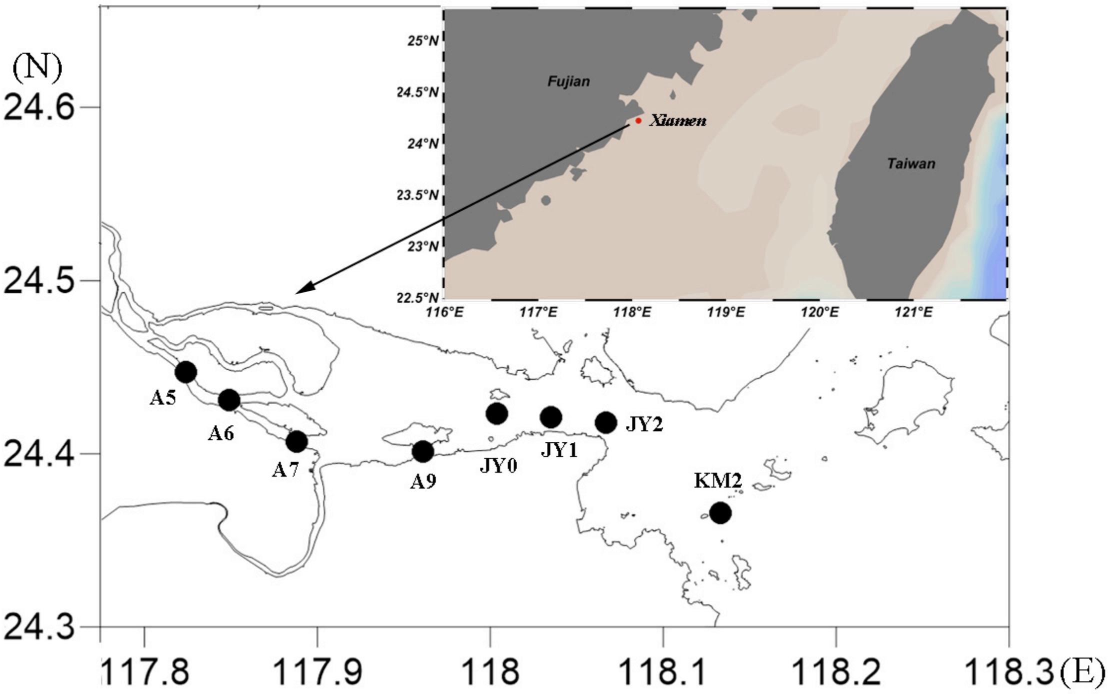

Table S1 Information of the sampling sites in Jiulong River Estuary.

Sampling sites	Latitude	Longitude	Sampling year	Sampling frequency
A5	24.447	117.824	2014	Every two months
A6	24.431	117.849	2014	Every two months
A7	24.407	117.888	2014	Every two months
A9	24.401	117.961	2014	Every two months
JY0	24.423	118.004	2014	Every two months
JY1	24.421	118.035	2014	Every two months
JY2	24.418	118.067	2014	Every two months
KM2	24.366	118.133	2014	Every two months

Table S2 Variations of the environmental factors across the sampling period in Jiulong River Estuary.

Sampling date	Temperature (◦C)	Salinity (PSU)	pH	NO3-N (μmol/L)	NO2-N (μmol/L)	NH4-N (μmol/L)	DTN (μmol/L)	DRP (μmol/L)	Chl-*a* (μg/L)	Bacteria (10^5cells/ml)
2014.02.12	12.5-15.1	0.4-28.4	-	51.1-224.9	3.6-14.9	18.1-241.8	153.5-563.4	1.74-9.70	1.65-19.85	7.87-47.87
2014.04.17	19.5-23.0	0.5-30.0	6.92-8.00	27.8-167.7	3.4-40.4	27.3-166.5	-	1.20-5.36	0.89-8.82	16.12-42.05
2014.06.04	25.3-27.7	0.0-27.0	6.91-8.09	-	-	36.7-84.3	186.0-368.9	2.35-7.00	0.47-4.02	10.00-43.41
2014.08.07	30.1-31.9	0.0-29.8	6.73-7.99	27.6-163.8	3.5-19.0	63.5-123.8	168.7-419.3	1.20-4.89	1.14-15.6	36.08-86.34
2014.10.10	26.0-26.4	2.7-31.1	7.02-8.08	14.8-178.7	6.9-50.1	12.7-53.3	104.9-370.6	0.05-1.10	0.36-10.95	9.75-45.53
2014.12.12	17.7-18.6	4.0-29.5	7.54-8.05	0.70-2.64	0.07-0.48	0.4-1.4	-	0.05-0.13	0.75-5.12	7.43-16.87

Table S3 Similarity within and between the three salinity groups±standard deviation (ANOSIM, P<0.001) as determined by Bray-Curtis similarity coefficient. F: freshwater; OM: oligohaline and mesohaline; PE: polyhaline and euhaline.

	Freshwater	OM	PE
Freshwater	45.6±16.4		
OM	27.7±12.6	38.8±12.9	
PE	11.1±10.4	26.0±11.3	40.1±13.0

Table S4 ANOSIM statistics tests of the groupings of communities according to salinity, temperature, and depth. Abbreviations: F, freshwater; OM, oligohaline and mesohaline; PE, polyhaline and euhaline. Community turnover was based on the Bray-Curtis distance.

	Total	
Grouping by	R	P
Salinity (global test)	0.660	<0.001
F versus OM	0.527	<0.001
OM versus PE	0.587	<0.001
F versus PE	0.917	<0.001
Temperature (global test)	0.252	<0.001
Depth (global test)	-0.066	>0.05

Table S5 Relative abundances of reads and OTUs in the abundant, intermediate and rare groups.

	Minimum	Maximum	Average	SD
Relative abundance of abundant OTU	2.597	18.382	11.617	3.114
Relative abundance of intermediate OTU	50.000	76.147	63.925	5.686
Relative abundance of rare OTU	11.010	44.286	24.459	6.240
Relative abundance of abundant reads	79.764	98.293	90.165	3.562
Relative abundance of intermediate reads	1.646	19.980	9.674	3.544
Relative abundance of rare reads	0.061	0.304	0.162	0.052

Table S6 BVSTEP and BIOENV analyses showing the correlations between environmental variables and the total/bimonthly collected community (which was randomly resampled from 14,825 to 100 sequences per sample).

Environment	BV-STEP factors	ρ	BIO-ENV factors	ρ
All samples	Salinity, Temperature, Chl *a*, Violaxanthin	0.589	Salinity	0.519
			Violaxanthin	0.368
February samples only	Salinity, Temperature, DRP	0.851	Salinity	0.797
			DRP	0.669
April samples only	Salinity, Temperature, Bacteria, NH_4, DRP, Microphytoplankton ratio	0.740	Salinity	0.629
			Temperature	0.219
June samples only	Salinity, Temperature, NH_4, Neoxanthin, Violaxanthin	0.863	Salinity	0.780
			DRP	0.755
August samples only	Salinity, Bacteria, Fucoxanthin, Alloxanthin, Diadinoxanthin	0.929	Salinity	0.915
			Diadinoxanthin	0.848
October samples only	Temperature, Neoxanthin	0.505	Temperature	0.366
			Chl *b*	0.339
December samples only	Salinity, Temperature	0.854	Salinity	0.932
			NH_4	0.770
